# Germ Theory: Medical Pioneers in Infectious Diseases, 2nd Edition

**DOI:** 10.3201/eid3007.231631

**Published:** 2024-07

**Authors:** Rachel N. Wofford

**Affiliations:** Tennessee Department of Health, Nashville, Tennessee, USA

**Keywords:** germ theory, infectious diseases, bacteria, antimicrobial resistance, public health, public health history, science pioneers

Robert P. Gaynes, professor at Emory University School of Medicine, has produced a second edition of *Germ Theory: Medical Pioneers in Infectious Diseases*, a chronicle of the evolution of germ theory told through stories of pioneers in science and medicine. The second edition adds new perspectives by expanding upon accounts of pioneers from the first edition of *Germ Theory*, such as Antony van Leeuwenhoek, Louis Pasteur, and Robert Koch. In addition, he introduces 3 pioneers of germ theory in the 20th Century, all personally interviewed by Gaynes (Figure). New chapters discuss the discoveries of HIV by Françoise Barré-Sinoussi and the connection between *Helicobacter pylori* and peptic ulcers by Barry Marshall. In addition, the book recounts the efforts of Anthony Fauci, from his early work in creating the President’s Emergency Plan for AIDS Relief in 2003 to more recently becoming the face of COVID-19 information in the United States during the pandemic.

**Figure:**
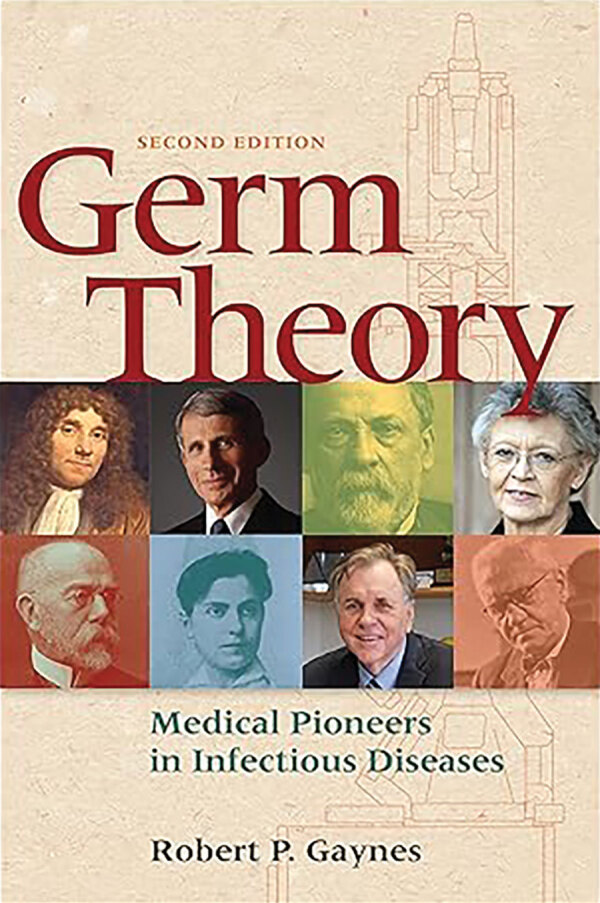
Germ Theory: Medical Pioneers in Infectious Diseases, 2nd Edition

Gaynes marvelously uses intersections between key historic figures and disease epidemics as guidestones on a walk through the history and evolution of germ theory, explaining current prevailing understandings among the general public, scientific community, or both, and reasons behind advancements and regressions over time in medicine and science. In each chapter, Gaynes dives deeply into the personality qualities, early influences, and lesser-known contributions of a prominent pioneer better known for a higher-profile contribution to medical science. Those accounts provide fresh context for familiar histories of medical landmarks. Gaynes also expands the context and breadth of discoveries by introducing persons who provided mentoring or otherwise supported the pioneering scientists, contradicted their work, or concurrently came to the same conclusions. 

Gaynes shares the frequent initial resistance of scientific and medical communities to discoveries. For example, he describes the persistence of the humoral theory of diseases into the early 18th Century because of the reputation of Galen of Pergamon, a 2nd Century Greek physician key to the foundation of Western medical education, who espoused that theory. Gaynes eloquently comments on this conflicted dynamic, stating that “major shifts in thinking often are products not just of the discovery but also of the age and culture.” In addition, by describing Fauci’s experiences working through 7 different presidencies, including during the COVID-19 pandemic, Gaynes provides insight into the connection between public health and politics at the highest levels of government. 

Gaynes weaves scientific concepts across chapters, effectively connecting historic events to modern-day challenges and guiding the reader through the effects and implications of the evolution of germ theory in a contemporary context. By making *Germ Theory* pertinent and understandable to readers in fields across science and medicine, Gaynes provides a wonderful tool to engage the next generation of public health professionals and reinvigorate those currently in the field, especially when public health workers are recovering from the challenges of the COVID-19 pandemic. Gaynes concludes the book with a call to future action to address emerging threats from antimicrobial resistance, vaccination hesitancy, and the effects of ongoing social vulnerabilities. 

